# Spectroscopic characterization of the warfarin drug-binding site of folded and unfolded human serum albumin anchored on gold nanoparticles: effect of bioconjugation on the loading capacity†

**DOI:** 10.1039/c8ra00006a

**Published:** 2018-02-16

**Authors:** Saba A. J. Sulaiman, Tanujjal Bora, Osama K. Abou-Zied

**Affiliations:** Department of Chemistry, Faculty of Science, Sultan Qaboos University P.O. Box 36, Postal Code 123 Muscat Sultanate of Oman abouzied@squ.edu.om; Nanotechnology Research Center, Sultan Qaboos University P.O. Box 17, Postal Code 123 Muscat Sultanate of Oman

## Abstract

Protein-conjugated gold nanoparticles (AuNPs) have recently shown promising applications in medicine, owing to their inertness and biocompatibility. Herein, we studied the spectroscopy of 25 nm diameter AuNPs, coated with human serum albumin (HSA) as a model drug carrier. The morphology and coating of the AuNPs were examined using transmission electron microscopy and dynamic light scattering. Resonance energy transfer from the sole tryptophan of HSA (Trp214) to the AuNPs indicates a single layer of protein coverage. Using fluorescein (FL) to probe the warfarin drug-binding site in HSA revealed an increase in the HSA–FL binding by ∼4.5 times when HSA is anchored on the nanoparticle surface, indicating a rise in the loading capacity. Femtosecond transient absorption measurements of the surface plasmonic resonance band of the AuNPs show three ultrafast dynamics that are involved in the relaxation process. The three decay components were assigned to the electron–electron (∼400 fs), electron–phonon (∼2.0 ps) and phonon–phonon (200–250 ps) interactions. These dynamics were not changed upon coating the AuNPs with HSA which indicates the chemical and physical stability of the AuNPs upon bioconjugation. Chemical unfolding of the warfarin binding site with guanidine hydrochloride (GdnHCl) was studied by measuring the spectral shift in the Trp214 fluorescence and the appearance of the Tyr fluorescence. Unfolding was shown to start at [GdnHCl] ≥ 2.0 M and is complete at [GdnHCl] = 6.0 M. HSA anchored onto the nanoparticle surface shows more resistance to the unfolding effect which is attributed to the stability of the native form of HSA on the nanoparticle surface. On the other hand, upon complete unfolding, a larger red shift in the Trp214 fluorescence was observed for the HSA–AuNP complex. This observation indicates that, upon unfolding, the HSA molecule is still anchored on the AuNP surface in which subdomain IIA is facing the outer water molecules in the bulk solution as well as the hydration shell rather than the core of the nanoparticle. The current study is important for a better understanding of the physical and dynamical properties of protein-coated metal nanoparticles, which is expected to help in optimizing their properties for critical applications in nanomedicine.

## Abstract 2

This work investigates the steady-state and ultrafast spectroscopy of bioconjugated gold nanoparticles and the implications on the protein binding activity and drug-loading capacity.

## Graphical abstract

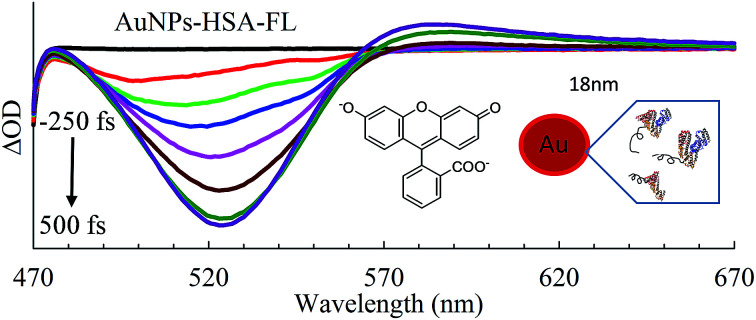

## Introduction

In recent years, the use of nanoparticles (NPs) conjugated with proteins has received much attention due to their exclusive optical properties that are size tunable.^1–4^ These properties are reflected in unique absorption signatures in the visible region.^3,4^ The conjugation process results in the formation of a layer from the protein that is covalently bound or adsorbed on the NP surface.^4–6^ The formation of this layer strongly depends on the size, surface charge, and the protein coating the NPs as well as the identity of the NPs.^7–9^ Besides stabilizing the NPs, this layer also reveals the biocompatible nature of the NP–protein complex, thereby allowing for further promising biological and chemical applications such as biosensing, imaging and drug delivery.^9,10^

The high surface-area to volume ratio of the NPs is advantageous for uploading drugs and enhancing their concentration. Metallic bioconjugated nanoparticles are known to be a common example for these characteristics, out of which gold NPs (AuNPs) in particular are heavily exploited due to their inert nature and high electron density.^11,12^ This, along with the non-toxic nature of gold, makes AuNPs a useful system that can be utilized in transporting and delivering a wide range of endogenous and exogenous substances in the biological medium.^12–14^

Coating the AuNPs with albumin proteins is useful due to their biocompatibility and their ability to bind many drug molecules.^14^ Among carrier proteins, human serum albumin (HSA) is considered one of the major transporter proteins in the blood plasma. It constitutes approximately half of the protein found in human blood.^15^ This protein with a single polypeptide chain and 585 residues is composed of three α-helical domains I–III, each containing two subdomains A and B (Fig. S1, ESI†).^16^ HSA contains two primary drug-binding sites.^16^ Site I (or the warfarin site) is situated in subdomain IIA and favors the binding of large heterocyclic and negatively charged compounds, whereas Site II (or the indole-benzodiazepine site) is located in subdomain IIIA and is the desired site for small aromatic carboxylic acids.^17–19^ In addition to the abundance of this protein in the circulatory system, it also possesses many physiological functions such as pH buffering and is used as a model protein in many biochemical applications.^20,21^

The sole tryptophan residue in HSA is located in Site I (Trp214) and plays a major role in the protein's spectroscopy.^18,19^ Photochemical excitation of Trp214 at 295 nm makes it a suitable fluorescent marker to study its local microenvironment. Upon adsorption of HSA on the NP surface, the change in the intrinsic fluorescence of tryptophan helps in understanding the influence of the NPs on the protein's binding properties.

**Scheme 1:**
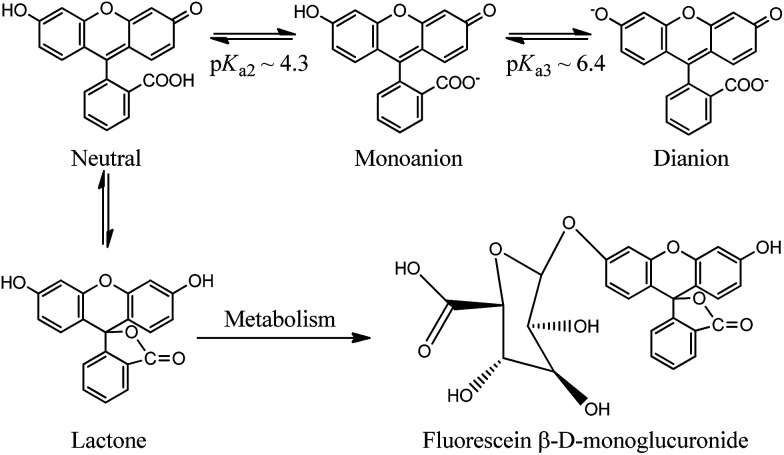
Prototypes of FL and the metabolism to fluorescein glucuronide.

Using a fluorescent molecule as an extrinsic probe should clarify the process of protein–ligand binding and interaction which is important in understanding the pharmacokinetics of drug and drug-like candidates. One of the widely used extrinsic labels in proteins is fluorescein (FL) which is characterized by a long wavelength absorption maximum near 490 nm with a high molar extinction coefficient, and high quantum yield of fluorescence.^22,23^ These properties are significant in biologically related applications that require a probe with spectroscopic signatures well-separated from those of biological samples. In addition to its versatile applications, FL is also used as a biological pH sensor to determine intracellular pH values (Scheme 1). The molecule displays a complex pH dependent equilibrium in which only the monoanionic and the dianionic forms are fluorescent.^22,23^ However a rapid metabolic mechanism to a weakly fluorescent conjugate (FL-monoglucuronide) was reported (see Scheme 1).^24,25^ In order to minimize the metabolic effect on FL, the molecule was used as a tracer after binding to HSA.^26,27^ We have recently characterized the binding properties of the HSA–FL complex using spectroscopic measurements in frequency and time domains, and found that FL moderately binds in the warfarin binding site of HSA.^28^

The importance of protein-conjugated AuNPs as drug carriers is inherent due to their target specificity and biocompatibility which are believed to reduce drug resistance in cells.^29,30^ These unique properties have led to promising applications of AuNPs in clinical treatments and disease diagnoses through high contrast AuNPs imaging, tracking and sensing.^31^ Extending the applications to include AuNPs as precursors to maintain and monitor the binding properties of protein–ligand systems would be useful. In this regard, any modification in the binding properties is important to examine. A direct effect on the kinetics of drug release is encountered if binding is too strong. On the contrary, it may contribute to inefficient drug transportation and distribution if binding is too weak. This can be achieved by studying the behavior of the protein with ligands/drugs under biologically relevant conditions when this protein is free in solution, compared to when the protein is adsorbed on the surface of NPs.

In the current work, we studied the binding mechanism of HSA on AuNPs of ∼25 nm diameter. We characterized the spectroscopic features of the albumin coated AuNPs in the absence and presence of FL as a ligand. Our results show that upon coating of the NPs with HSA, the binding strength of Site I toward FL increased 4.5 times, thereby suggesting a better loading ability of the protein. The AuNPs–HSA–FL system is characterized by femtosecond transient absorption spectroscopy which indicates a stable confirmation for the AuNP-bioconjugated system. Finally, chemical unfolding point to the stability of the wild-type form of HSA on the nanoparticle surface.

## Experimental section

### Materials and chemicals

HSA (essentially fatty acid free) was obtained from Sigma. Concentration of HSA in de-ionized (DI) water and in AuNPs was prepared using its listed molecular weight of 66.5 kDa, and the final concentration was checked by comparing the measured absorbance in DI water with the published value (optical absorbance at 279 nm = 0.53 (1 g L^−1^)).^32^ FL, l-tryptophan and l-tyrosine were purchased from Sigma-Aldrich. Guanidinium chloride (GdnHCl, ≥99%) was received from VWR Chemicals.

### Synthesis of citrate capped colloidal AuNPs

The AuNPs were synthesized in aqueous medium as reported previously using a modified reduction Turkevitch method.^33^ Briefly, an aqueous solution of 2.0 ml of 5 mM gold chloride hydrate (HAuCl_4_·H_2_O) was added to 50.0 ml DI water in a conical flask and the mixture was heated to boil. Upon boiling, 2.8 ml of 25 mM trisodium citrate (TSC, Na_3_C_6_H_5_O_7_·2H_2_O) was added drop wise under constant stirring. The conical flask was dipped immediately in an ice-cold water bath in order to quench the reaction as soon as the desired red color was attained and remained unchanged. The colloidal AuNPs were then stored in refrigerator for further use. The AuNP concentration was estimated to be 7.81 × 10^−10^ M by measuring the absorbance of the surface plasmonic resonance (SPR) peak, and using the published extinction coefficients.^34^

### Preparation of the AuNP–HSA system

4 μM of HSA was dissolved directly in the prepared AuNPs, following previously reported procedures.^35–37^ The stock solution was stirred vigorously for 2 h and incubated at room temperature for 6 h. A set of different AuNPs concentrations was then prepared by diluting the stock solution using an aqueous solution including the same HSA concentration. A second set of experiments was performed by changing the HSA concentration and maintaining the AuNPs concentration at 7.81 × 10^−10^ M. The absorption and fluorescence measurements were then performed and repeated after 12 h, and no significant differences were observed. No further separation technique was applied to extract the excess protein in the bioconjugated nanoparticles used in this study in order to avoid any disruption in the original conformation of the protein as well as the AuNPs.^38^

### Preparation of the AuNP–HSA–FL system

A stock solution of FL was prepared in dioxane (5 mM) and then diluted to different concentrations in aqueous solutions containing HSA and AuNPs. The total volume of dioxane was less than 5%. A ratio of dioxane : H_2_O (v/v) above 10 : 90 was shown to behave like pure water.^39^ The concentration of FL was gradually increased while maintaining the concentration of HSA and AuNPs fixed at 4 μM and 7.81 × 10^−10^ M, respectively. The prepared solutions were equilibrated for 6 h before taking the measurements. The measurements were then repeated after 12 h and no significant change was observed.

### Instrumentation

Absorption spectra were obtained with an Agilent 8453 Diode Array UV-vis spectrophotometer. Fluorescence spectra were recorded on a Shimadzu RF-5301 spectrofluorophotometer. The fluorescence spectra were corrected for the difference in optical density at the excitation wavelength and inner-filter effect using the following equation:^40,41^

1 *F*_corr_ = *F*_obs_ × antilog((OD_ex_ + OD_em_)/2)

where *F*_corr_ and *F*_obs_ are the corrected and observed fluorescence intensities, respectively, and OD_ex_, OD_em_ are the optical density at both the excitation and emission wavelengths, respectively. Time-resolved fluorescence measurements were performed using a Time-Master fluorescence lifetime spectrometer obtained from Photon Technology International. Excitation was at 280 and 295 nm using light emitting diodes. The system response time as measured from the scattered light was estimated to be approximately 1.5 ns. The measured transients were fitted to multi-exponential functions convoluted with the instrument response function (IRF). The fit was judged by the value of the reduced chi-squared (*χ*^2^). The experimental time resolution (after deconvolution) was approximately 100 ps, using stroboscopic detection.^42^

High-resolution transmission electron microscopy (TEM) measurements on the AuNPs were performed using a JEOL, JEM-2100F microscope at an operating voltage of 200 kV. A drop of aqueous dispersion of AuNPs solution was placed on carbon coated copper grids and the samples were left to dry at room temperature. Dynamic light scattering (DLS) was performed using Cilas Nano DS to evaluate the hydrodynamic diameter of the NP–HSA at room temperature. Surface charge of the AuNPs–HSA at room temperature was obtained through zeta potential measurements using NICOMP 380 ZLS. The microchemical analysis was performed using an energy dispersive spectrometer (EDS) EX-24063 JGT, JOEL.

The ultrafast transient absorption measurements were performed using a femtosecond laser setup that was previously described in detail.^43^ Briefly, pump and probe pulses were obtained using a regenerative amplified Ti:sapphire laser (Libra, Coherent). The Libra generates compressed laser pulses (70 fs pulse width) with output of 4.26 W at a repetition rate of 5 kHz and centered at 800 nm. The output beam was split into two parts. The major portion of the output pulse was used to pump a Coherent OPerA Solo (Light Conversion Ltd.) optical parametric amplifier to generate spectrally tunable light spanning the range 240–2600 nm and is used to generate the pump beam at 460 nm. The remaining small portion of the laser output was focused on a sapphire crystal to generate a white light continuum in the range 430–800 nm which is used as the gate pulse in a Helios transient absorption spectrometer (Ultrafast Systems, LLC). The probe light was measured by a fiber optic that is coupled to a multichannel spectrometer with a CMOS sensor in the range 350–850 nm. Chirp in the white light continuum probe was minimized by using parabolic mirrors. Rotational contribution to the overall excited state decay kinetics was removed by depolarizing the pump beam using a depolarizer (DPU-25, Thorlabs). The pump pulse was attenuated to ∼150 nJ in order to avoid multiphoton excitation.^44–46^

The pump and probe pulses were focused on the sample and the temporal delay of the probe pulse was varied using a computer-controlled optical delay stage (up to 5 ns with a shortest delay step of 20 fs). Kinetic traces at appropriate wavelengths were assembled from the time-resolved spectral data. Surface Xplorer software (supplied by Ultrafast Systems) was used for data analysis. The instrument response was estimated to be ∼120 fs as determined from the coherent artifact signal of the pure solvent (dashed line in Fig. 8). The wavelength dependence of the signal from the solvent (Fig. S6, ESI†) was used to correct time-zero of the time-resolved absorption data.

The samples for the pump–probe measurements were prepared in 2 mm fused silica cuvettes (Spectrocell Inc.) and were stirred during the experiment to avoid photodegradation. All measurements were conducted at 22 ± 0.5 °C. The steady-state absorption spectra of the samples were compared before and after the experiments and no change was observed in the absorbance and the spectral shape, indicating no photodegradation effect due to the laser pulses.

## Results and discussion

### AuNPs characterization

**Fig. 1:**
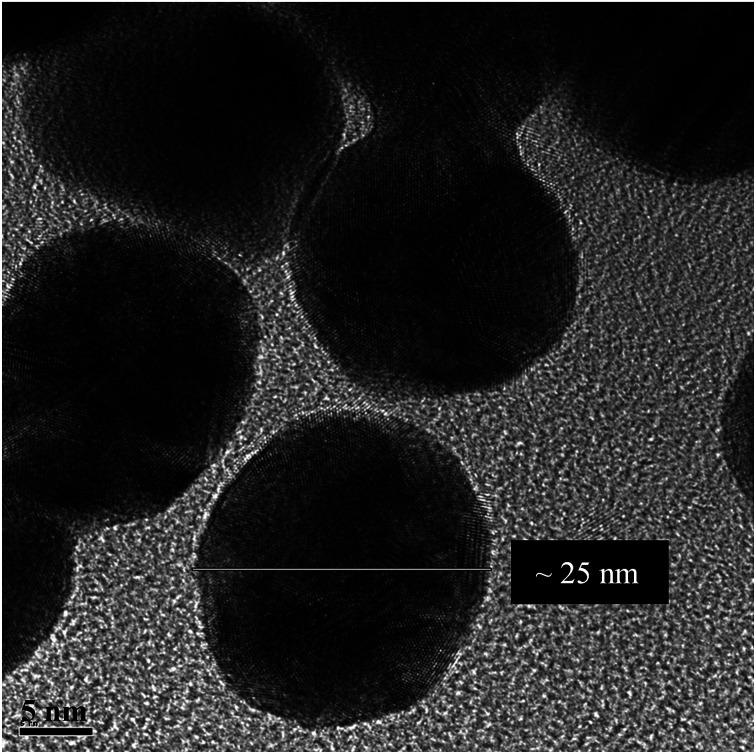
TEM image of bare AuNPs at pH 7.2.

**Fig. 2:**
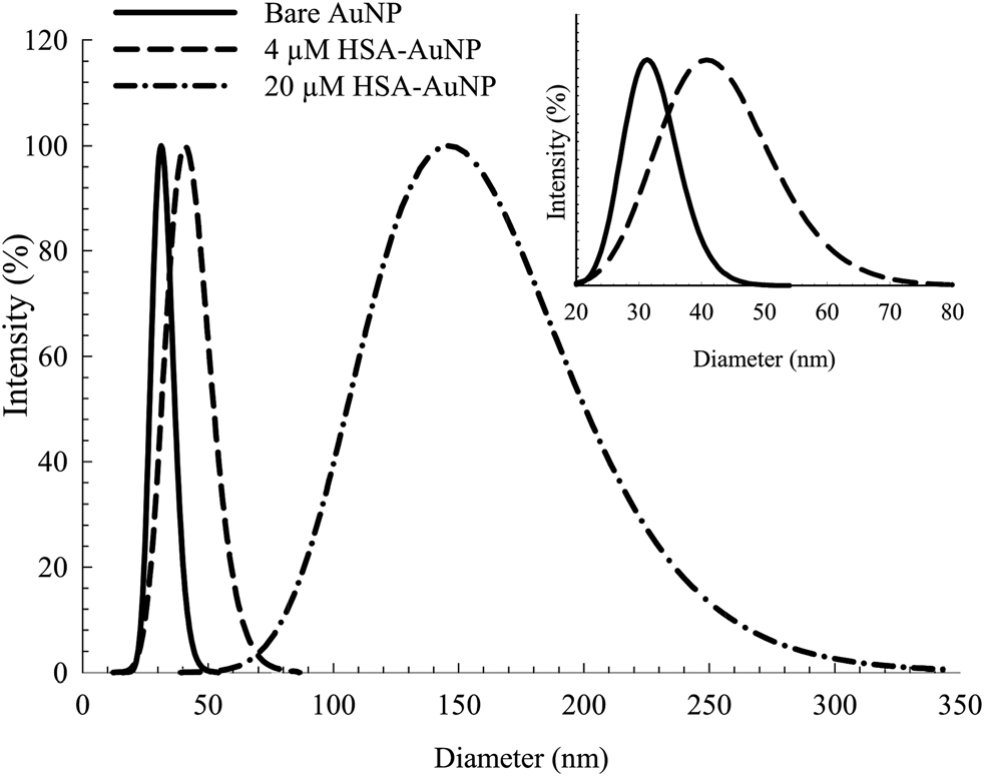
DLS spectra of bare AuNP and albumin coated AuNP with 4 μM and 20 μM HSA. Inset: expanded scale for the bare AuNP and AuNP coated with 4 μM HSA.

Fig. 1 shows the TEM of the bare NPs. The image reveals the nearly spherical shape of the AuNPs, with an average diameter of ∼25 nm. Fig. S2, ESI† shows the TEM of mono-dispersed nanoparticles on a larger scale. The EDS (Fig. S3, ESI†) confirms the presence of C, N, S, O and Au. The detected Cu is from the TEM grid. DLS and zeta potential measurements were used to study the NPs–protein interaction to reveal the degree of surface coverage and the influence of HSA on the hydrodynamic radius of the AuNPs. This was achieved by treating the AuNPs with an aqueous solution containing 4 μM of HSA. Fig. 2 shows the DLS measurements of the bare AuNPs and the AuNPs–HSA complex. The protein anchored on the surface of the AuNPs is expected to produce considerable changes in the hydrodynamic radius of the AuNPs–HSA complex, compared to bare AuNPs. The results indicate that the hydrodynamic diameter of the AuNPs is ∼30 nm and that of the bioconjugated nanoparticles is ∼41 nm. Taking into consideration that the DLS measurements were conducted in aqueous solution, the hydrodynamic diameter then reflects the increase in thickness due to water layer.^38^ Sen *et al.* reported that the DLS measurement of pure HSA at pH 7 yields a hydrodynamic diameter of 8 nm.^47^ Sharma and Ilanchelian also reported the increase in the hydrodynamic radii of AuNP in the presence of HSA from an initial value of 24 ± 2 nm to 31 ± 2 nm.^9^ Our results are thus consistent with the reported values.

On the other hand, the TEM measurements were performed on dry samples. However, the significant size increase upon conjugation is a consequence of the albumin binding (7 nm in thickness, considering the average size of an HSA macromolecule).^16^ The estimated increase in diameter (∼11 nm) falls within the appropriate range of a mono-dispersed layer of HSA coating. For a particle size of 25 nm in diameter, an estimated 150–200 molecules of HSA are needed for one layer coverage.^36,48^

Increasing the HSA concentration to 20 μM shows an increase in the AuNPs–HSA diameter to ∼145 nm (Fig. 2, inset), indicating multilayer coverage of each AuNP with HSA molecules and no signs of agglomeration since the DLS spectra obtained is typical of bioconjugated AuNPs. This suggests that the AuNPs are well dispersed in the medium. The results show the possibility of multi-layer capping with HSA around the NPs, while preserving the identity of the AuNPs.^36^

Zeta potential (*ζ*) measurements reveal vital information regarding the stability of the NPs dispersion. This is usually reflected by the balance created between the attractive and repulsive forces of the NPs, thereby, providing information about the way the NPs approach each other. The positive values obtained for the bare AuNPs and the albumin capped NPs, respectively, imply that they are stable with no clear affinity for agglomeration.

### Spectroscopic characterization of albumin conjugated AuNPs

**Fig. 3:**
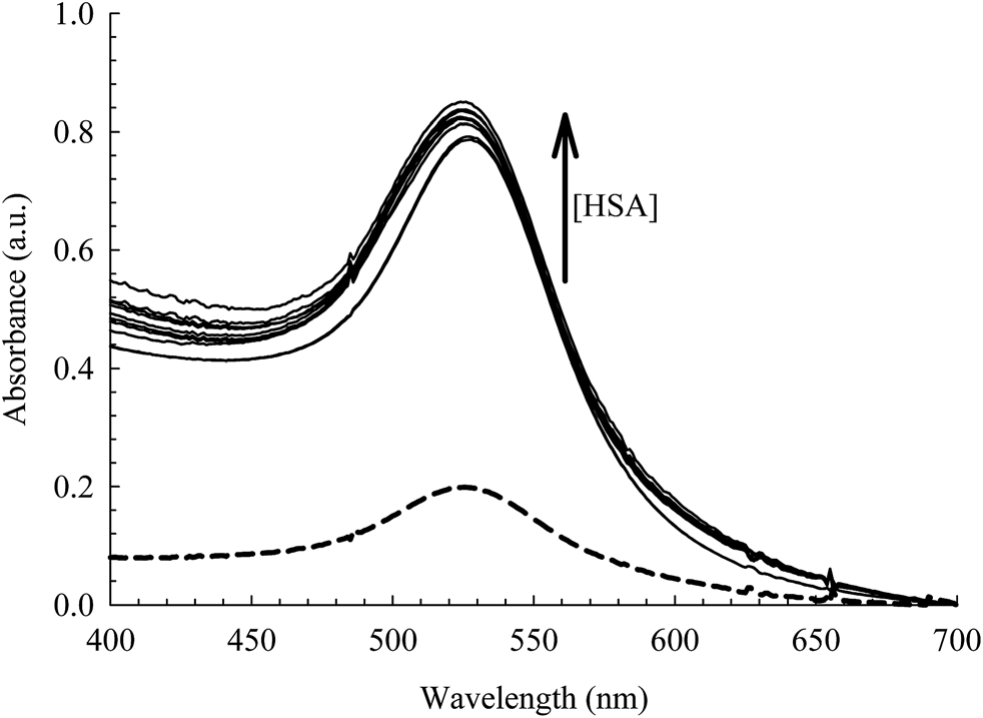
Absorption spectra of the AuNPs [0.78 nM] at different concentrations of HSA (0.0–50 μM). Dashed curve is when [HSA] = 0.0 M.

The absorption spectra of the citrate capped AuNPs in the absence and presence of bioconjugated HSA are displayed in Fig. 3. The AuNPs exhibit a distinct SPR band that is characteristic of nanoparticles at 520 nm and is an indication of an aggregation free environment.^9,13,29,49^ Upon uploading the NPs with HSA (2 μM), a significant rise in the intensity of the SPR band was observed with no shift in *λ*_max_ or rise in the baseline (see Fig. 3). Subsequent addition of HSA (>4–50 μM) results in a slight increase in the SPR band intensity with a slight blue shift (2–3 nm). This indicates no flocculation of the AuNPs, since a dramatic change in the optical properties of the SPR band was not observed.^9,29^ The change in intensity, therefore, can be interpreted by the association of HSA on the surface of the NPs, leading to an efficient energy transfer from HSA fluorescence to AuNPs absorption as will be discussed below. The blue shift of the SPR band when the concentration of HSA is increased beyond 4 μM can be correlated to the increase in the surface charge density on the AuNP surface due to multilayer coverage by the protein. This will lead to a smaller volume for the free electrons on the nano-metal surface, thus an increased free electron density and a higher plasmon frequency (shorter wavelength).^50,51^ Although this change is very small, we used 4 μM of HSA in our subsequent study in order to avoid this effect.

**Fig. 4:**
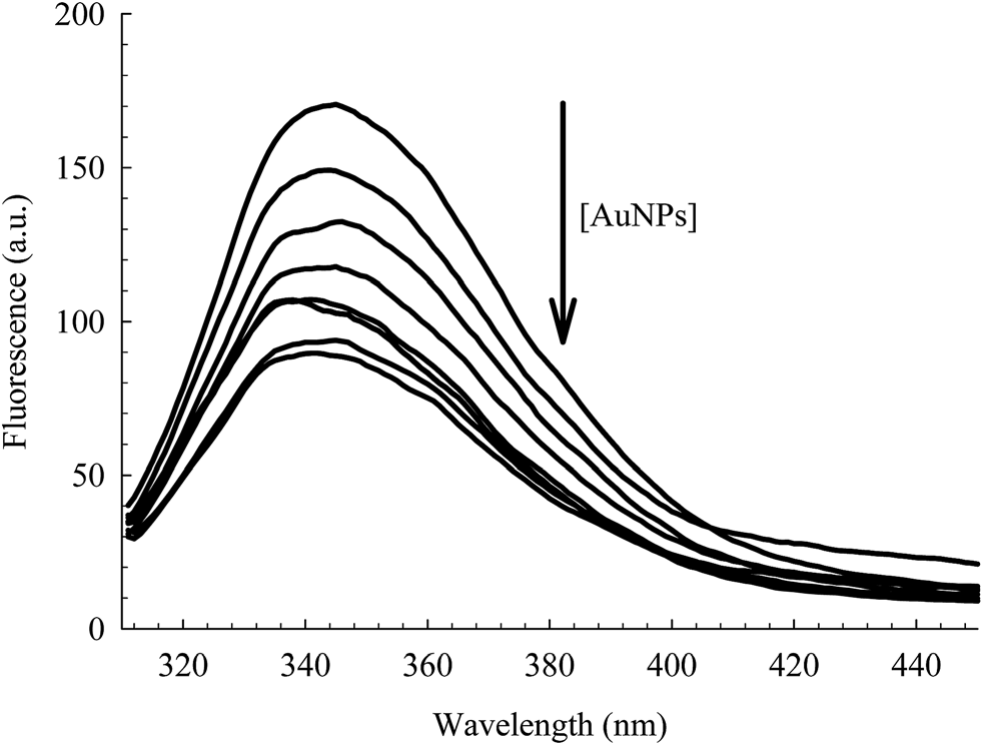
Fluorescence of Trp214 in 4 μM HSA at different concentrations of AuNPs (0.00–0.78 nM). *λ*_ex_ = 295 nm.

The intrinsic fluorescence of HSA is an informative tool to detect the presence of a ligand near the Trp214 residue.^40,52^ As seen in Fig. 4, the fluorescence intensity of HSA is progressively quenched upon increasing the AuNPs concentration.^3^ The decrease in the Trp214 fluorescence intensity, along with the increase in the absorbance of the conjugated NPs, is due to the change in the local environment around the protein that is adsorbed on the surface of the NPs.^3,6,38,53^ Applying the Förster's resonance-energy transfer (FRET) theory, we estimated the apparent distance between Trp214, as the donor, and AuNP, as the acceptor (details in ESI†). Both donor and acceptor must be in close proximity and share certain spectroscopic properties.^48,54^ This is evident in the spectral overlap between the absorption spectrum of AuNP and the fluorescence spectrum of HSA (Fig. S4, ESI†). We calculated an apparent distance between Trp214 and AuNP (*R*_DA_) to be ∼18 nm (Table S1, ESI†). This result seems to fall outside the acceptable range of the FRET regime (7 nm), however it is important to consider the radius of the AuNP, as well as the hydration shell within the AuNPs–HSA system. According to our TEM (Fig. 1) and DLS (Fig. 2) results, the average radius of the AuNPs is ∼12.5 nm. Accordingly, the apparent distance between Trp214 and the AuNP-core should be ∼5.5 nm.

The fluorescence decay transients of HSA in the absence and presence of AuNPs are shown in Fig. S5, ESI.† Two lifetime components were extracted from each decay transient that reflect the dynamics of Trp214. No change in the average lifetime (*τ*_av_ = 5.60–5.65 ns, Table S2, ESI†) was observed which indicates the dominant role of static quenching in the AuNPs–HSA system.^40^ The two lifetime components of native HSA are typical of an HSA molecule solvated in water.

**Fig. 5:**
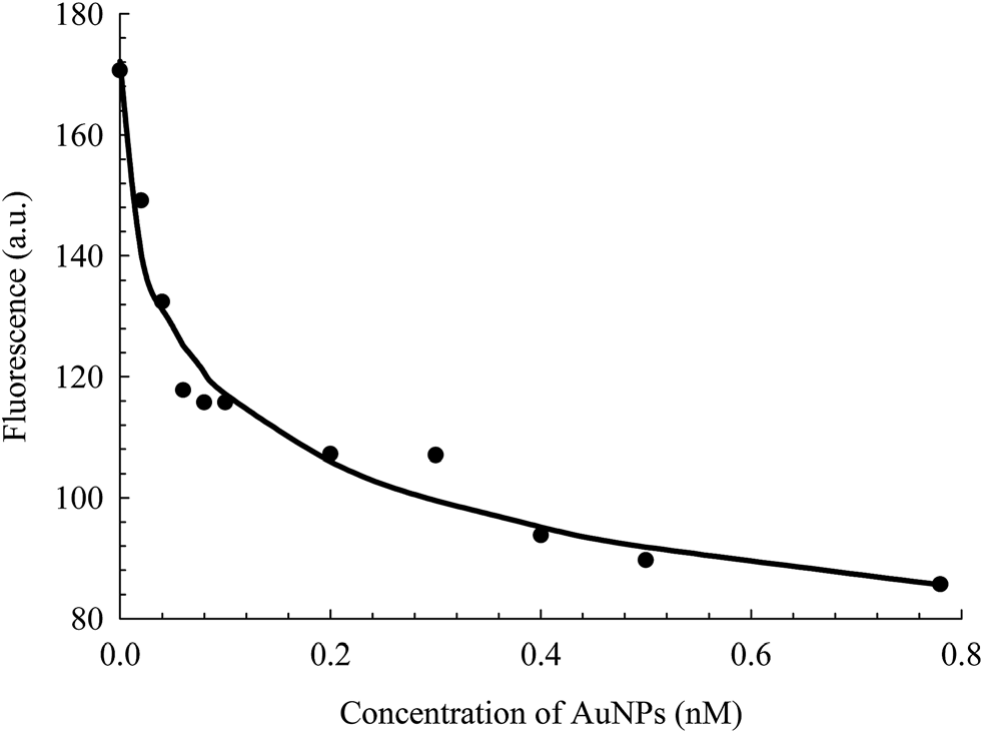
Fluorescence change of HSA as a function of the concentration of AuNPs, extracted from the data in Fig. 4. The solid line is the best non-linear regression fit to eqn (2).

The binding strength of HSA on the surface of the AuNPs can be estimated using a binding isotherm that is represented by eqn (2):^55^2
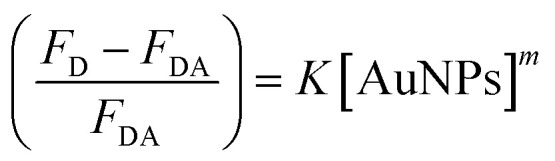
where *F*_D_ and *F*_DA_, respectively, are the fluorescence intensity of the donor alone (HSA) and of the donor/acceptor mixture (HSA–AuNPs). *K* and *m* are the equilibrium binding constant and the protein binding cooperativity, respectively. The latter is known as Hill coefficient that is used as a measure of the protein association on the surface of the NPs.^3,9^Fig. 5 shows the change in the HSA fluorescence for different AuNPs concentrations. The best fit to eqn (2) yields values of *K* = (1.60 ± 0.24) × 10^9^ M^−1^ and *m* = 0.98 ± 0.10. The value of *m* (close to 1) indicates that binding between the individual HSA molecules and the surface of the AuNPs is independent of other protein molecules present at the surface.^3,9^

### Fluorescein binding to HSA and HSA-conjugated AuNPs

FL binds to the HSA macromolecule at Site I as we recently reported.^28^ The mode of binding of FL was found to be similar to that of the anticoagulant drug warfarin. For small molecules that bind independently to a set of equivalent sites in a macromolecule, eqn (2) can be used to calculate *K* and *m*, where *m* here reflects the number of equivalent binding sites in the macromolecule.^40^ For the purpose of comparison, we converted eqn (2) into a linear equation as shown below:^28,56^3
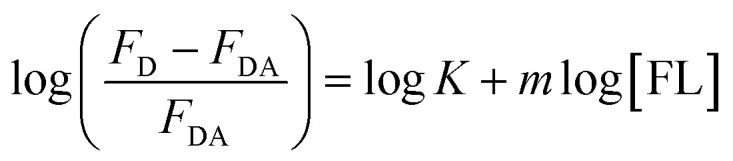


We have shown recently that binding of FL in Site I of HSA can be characterized using eqn (3), where the *m* value is 1 for relative concentrations of up to 1 : 1.5 ([HSA] : [FL]).^28^ Upon increasing the FL concentration, the value of *m* increases due to binding of more than one FL molecule in HSA. The results reflect the loading capacity of HSA to bind more than one ligand which in important for the protein as a major drug carrier.

**Fig. 6:**
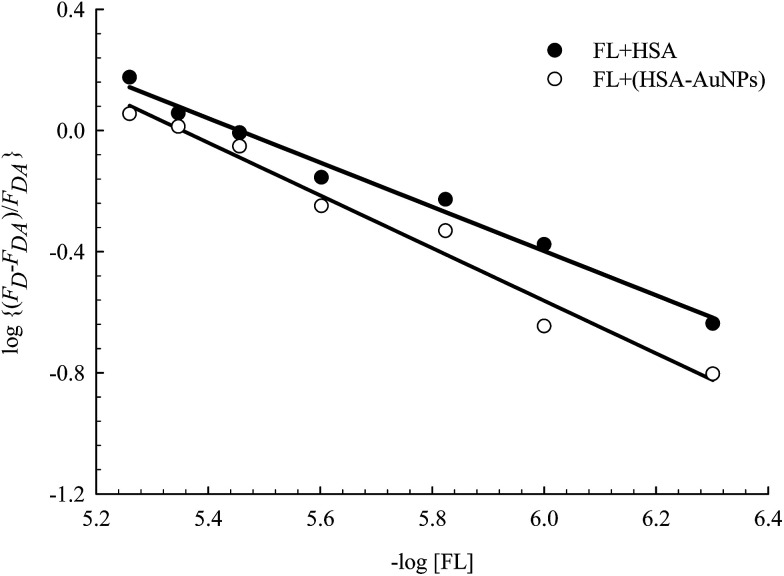
Effect of FL (0–30) μM on HSA fluorescence in the absence and presence of AuNPs. Solid lines represent the linear behavior according to eqn (3). Concentration of HSA was 4 μM and that of AuNPs was 0.78 nM.

Fig. 6 displays the change in log((*F*_D_ − *F*_DA_)/*F*_DA_) as a function of log[FL] for the binding of FL in HSA and HSA-coated AuNPs. As shown in the figure, a noticeable change was observed when FL binds to HSA-coated AuNPs, compared to its binding in HSA free in solution. The equilibrium binding constant (*K*) for FL/HSA is estimated to be (1.0 ± 0.12) × 10^4^ M^−1^, whereas *K* for FL/HSA–AuNPs is (4.60 ± 0.19) × 10^4^ M^−1^. The *m* value in both systems was 0.90 ± 0.10, indicating a molar ratio of 1 : 1 (FL : HSA). Coating of AuNPs with HSA thus enhances the ligand binding, *i.e.* more loading capacity. This can be correlated to the large surface area of the NPs that makes the HSA macromolecules more available for ligand binding.

### Femtosecond-nanosecond transient absorption

**Fig. 7:**
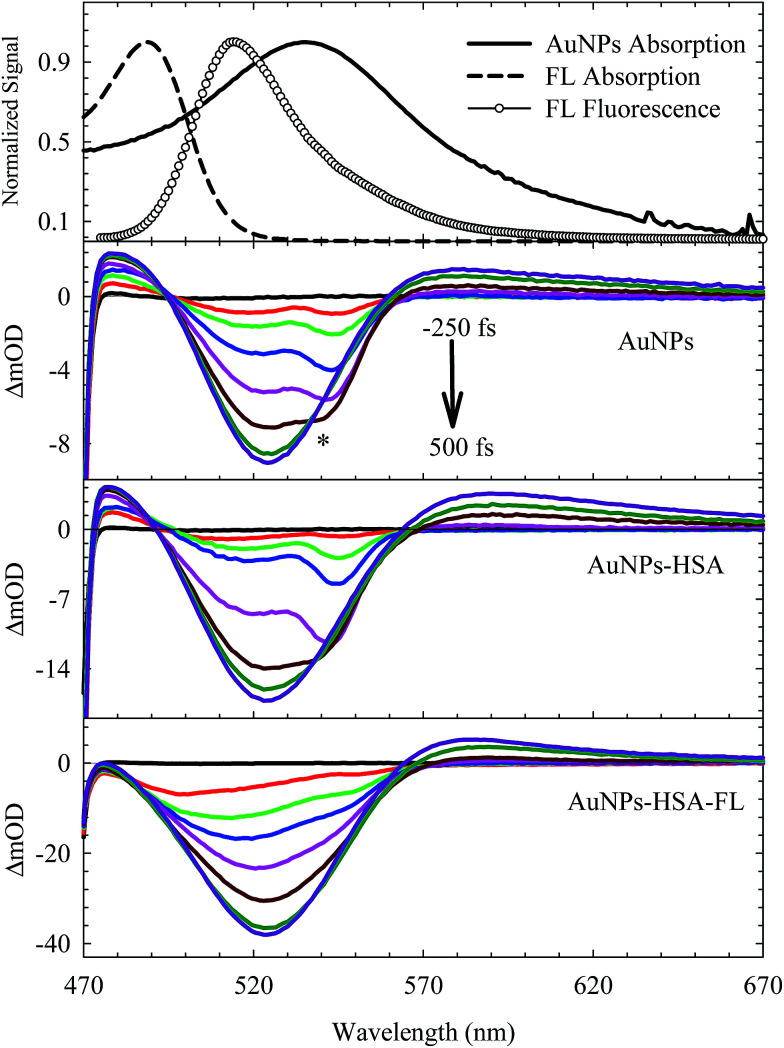
Femtosecond transient absorption spectra of different systems as indicated in the graph. The dynamics were recorded during the first 500 fs immediately after photoexcitation at 460 nm. * represents Raman scattering due to the solvent. Steady-state absorption and fluorescence spectra are shown at the top.

The electronic transition and relaxation dynamics of AuNPs were examined in the femtosecond to nanosecond time range by recording the absorbance change in real time. Fig. 7 shows the spectra for different systems during the initial period of 500 fs after excitation at 460 nm. The transients for the bare AuNPs show positive absorbance wings around the bleach peak centered at ∼525 nm. The two wings exhibit an instantaneous build up within a few hundred femtoseconds, then decays with a time constant of about 2.0 ps. The two photoinduced absorbance bands are the results of the change in the dielectric constant and the increase in the electron temperature of the NPs which result in broadening of the SPR band.^45,57^

**Fig. 8:**
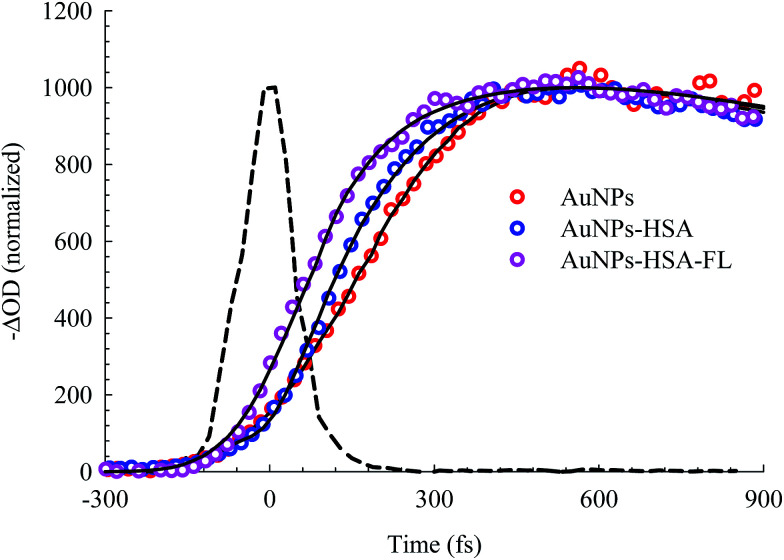
Femtosecond dynamics of AuNPs, and its conjugated systems, derived from the transient absorption spectra shown in Fig. 7. The dashed line represents the system response measured from the Raman scattering of the solvent.

**Fig. 9:**
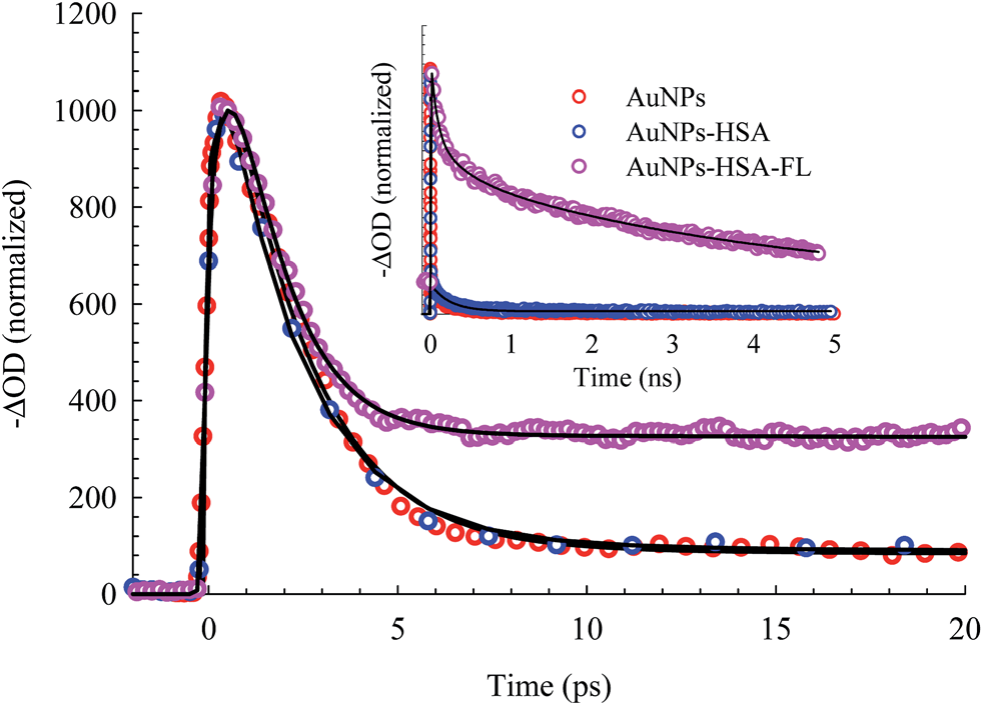
Picosecond to nanosecond dynamics of AuNPs, AuNPs–HSA, and AuNPs–HSA–FL systems, derived from the transient absorption spectra.

The bleach band at 525 nm represents the temporal change in the SPR peak (see the steady state absorbance in the top segment of Fig. 7). More insight into the dynamics of this peak is shown in the transients in Fig. 8 and 9. The transient absorption spectra in Fig. 8 show an increase in the bleach intensity during the first 500 fs period. The fits in Fig. 8 show the buildup in intensity as a time constant of ∼400 fs for the bare AuNPs which is assigned to electron–electron interaction. This interaction gets slightly shorter in the bioconjugated AuNPs with HSA and HSA/FL. The fast rise is followed by a decay component of about 2.0 ps that represents the electron–phonon interaction.^45,46^ The final relaxation dynamic is due to phonon–phonon interaction and takes place is hundreds of picoseconds (200–250 ps from the fits in Fig. 9).

Due to the presence of FL, additional slow dynamics are encountered in the transient signal of AuNPs–HSA–FL (see Fig. 9). FL absorbs strongly at 460 nm and its emission strongly overlaps with the AuNPs absorption, as shown in the upper segment of Fig. 7. Excitation of FL in the 460 nm region exhibits an instantaneous rise, followed by two long decay components for the monoanion (1.36 ns) and the dianion (4.92 ns) species.^28,57^

The transient absorption curves in Fig. 7 (last segment) show the effect of the presence of FL in the sample in which the blue wing (transient absorption in the region 470–490 nm) is suppressed. Transient absorption of FL shows only a bleach recovery that overlaps with the positive absorbance of the AuNPs (Fig. S6, ESI†). Accordingly, the spectra in Fig. 7 for AuNPs–HSA–FL are mixtures of those of AuNPs–HSA and FL.

The transient absorption results point to the physical and chemical stability of the AuNPs upon bioconjugation. From the DLS and TEM results shown above, HSA forms a coverage layer around the NP where the protein is in close proximity to the electron cloud of the NP. The FRET results also indicate an efficient energy transfer from HSA to the NPs, upon HSA excitation at 295 nm, where the static contribution dominates the quenching mechanism. Since the energy relaxation pathway in the AuNPs is completed through phonon–phonon interactions with the encapsulating material or surrounding medium, the measured unchanged ultrafast dynamics of the AuNPs upon bioconjugation with HSA, and in the presence of FL, confirm the dominant role of the static quenching, in addition to the preserved structural identity of the AuNPs. It is important to mention here that HSA is transparent at 460 nm (the excitation energy in the transient absorption experiments). Many organic materials have shown to act as a thermal reservoir, hence affecting the relaxation dynamics, in which photoluminescence can be detected from these materials in encapsulated NPs.^58^ In the present system, we did not observe any significant change in the FL fluorescence in the AuNPs–HSA–FL complex. This observation can be a consequence of the presence of self-quenching mechanism in FL, which was reported to be very efficient even at low concentrations of FL.^59^ Self-quenching of the FL fluorescence is attributed to the very small Stokes shift between the absorption and fluorescence spectra of FL, as shown in the upper segment of Fig. 7.

### Chemical unfolding of HSA

**Fig. 10:**
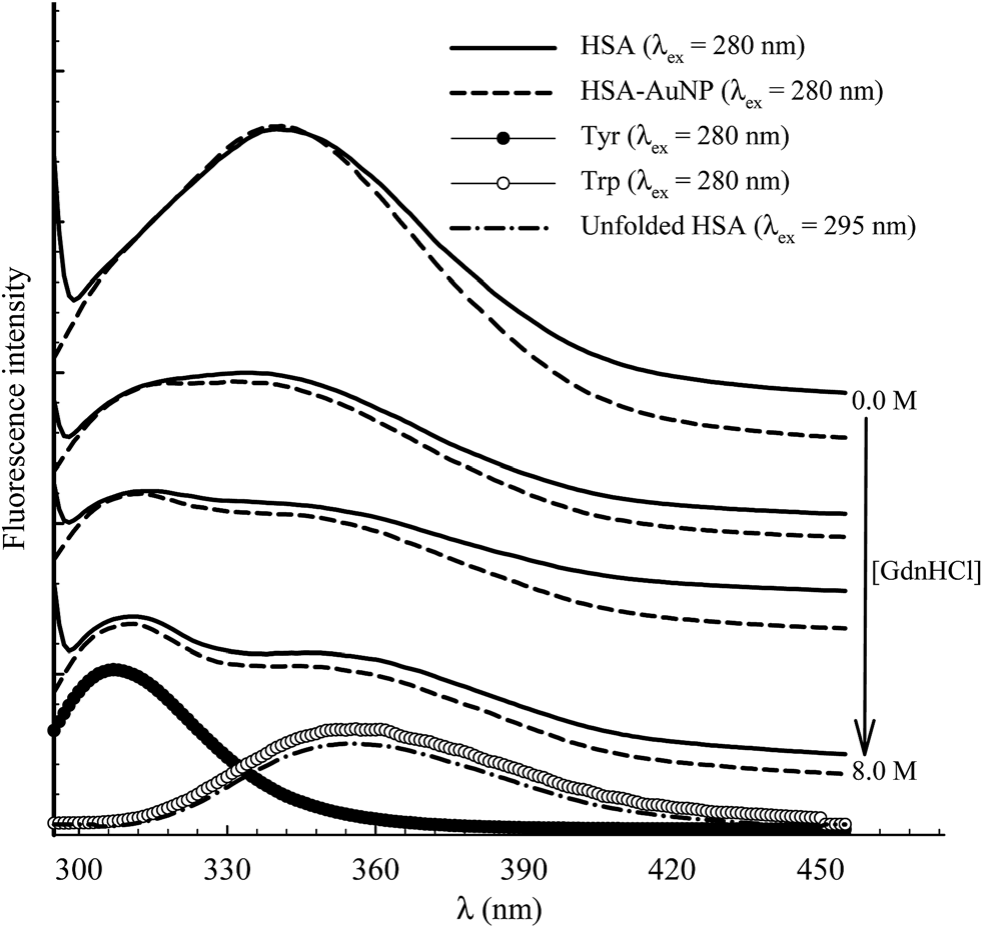
Selected fluorescence spectra of 4 μM HSA (solid lines) and HSA–AuNP (dash lines) during the process of chemical unfolding using GdnHCl (concentration as indicated in the graph) after excitation at 280 nm. The corresponding spectra of aqueous Tyr and Trp are shown at the bottom. Fluorescence spectrum of unfolded HSA ([GndHCl] = 8.0 M) is shown at the bottom after excitation at 295 nm.

The function of HSA, like any other protein, is associated with its native structured state. When the structure is altered, the specific functions carried out by the protein are endangered. Here, the unfolding of subdomain IIA is studied by measuring the change in the Trp214 fluorescence of HSA as a function of anhydrous GdnHCl concentration. Fig. 10 shows the results for selected GdnHCl concentrations in a comparative manner for HSA in the absence and presence of AuNPs. The study is aimed at testing the stability of the native form of the protein when anchored on the nanoparticle surface.

No apparent change was observed when the GdnHCl concentration is increased up to 2.0 M. Further increase in [GdnHCl] causes a reduction in the fluorescence intensity and a slight red shift. At the same time, a new peak at 308 nm starts to develop as the concentration of GdnHCl increases. We have previously shown that the shift in the Trp214 peak is due to unfolding of HSA in which the Trp214 residue is exposed to aqueous medium.^60^ A decrease in the fluorescence intensity, upon exposure of Trp214 to water, is due to solute–solvent interaction that enhances nonradiative depletion of the excited state energy. The new peak at 308 nm is due to fluorescence from tyrosine (there are 18 Tyr residues in HSA) in which unfolding of HSA reduces the internal quenching effect on the Tyr fluorescence. Tyrosine fluorescence is quenched inside the native form of HSA as a result of being affected by the presence of nearby amino acids, particularly Trp214. Assignment of the HSA fluorescence peak at 308 nm to Tyr is confirmed by comparing its spectral position with that of free Tyr in aqueous solution (Fig. 10).

**Fig. 11:**
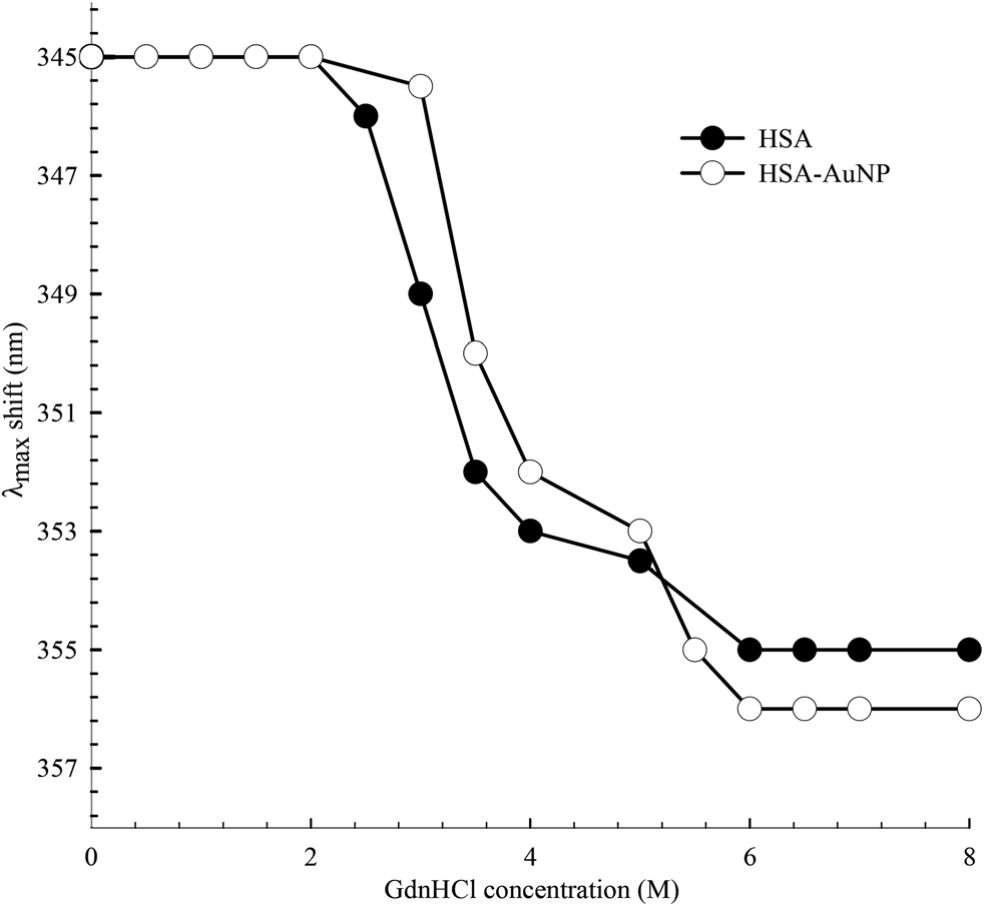
Spectral peak shift of Trp214 fluorescence as a function of GdnHCl concentration for HSA in the absence and presence of AuNPs. *λ*_ex_ was 295 nm.

The peak position of the Trp214 fluorescence reflects its local environment. In native HSA, the peak position is at 345 nm, indicating a partially buried residue.^61^ In order to accurately determine this position during the unfolding process, we excited HSA at 295 nm where only Trp absorbs. The peak shift as a function of GndHCl concentration is displayed in Fig. 11 and the spectra are shown in the ESI (Fig. S7†). For HSA free in solution and anchored on the AuNPs, unfolding starts at [GdnHCl] ≥ 2.0 M and is complete at [GdnHCl] = 6.0 M. The latter is confirmed by comparing the peak position of unfolded HSA with that of Trp in aqueous medium (Fig. 10), particularly when HSA was excited at 295 nm. It is clear from Fig. 11 that HSA anchored on the nanoparticle surface shows more resistance to the unfolding effect at lower GdnHCl concentrations, compared to HSA free in solution. This may be due to the stability of the native form of HSA on the nanoparticle surface. In the same context, the presence of AuNPs was shown to improve the thermal stability of the native form of BSA at pH 6.^62^ On the other hand, upon a complete unfolding, a larger red shift was observed for the HSA–AuNP complex. This observation indicates that, upon unfolding, the HSA molecule is still anchored on the AuNP surface in which subdomain IIA is facing the outer water molecules in the bulk solution as well the hydration shell rather than the core of the nanoparticle.^63^

## Conclusions

We investigated the spectroscopy of HSA-bioconjugated AuNPs as a model drug carrier in order to clarify the effect of the protein on the properties of the NPs and the stability of the protein structure on the metal surface. The morphology of the AuNPs was confirmed by TEM, whereas a single-coating layer by HSA was observed in the DLS results. The FRET results indicate an efficient energy transfer from the sole tryptophan residue in HSA to the AuNPs, where static quenching dominates the mechanism of the energy transfer. The binding strength of FL, as a fluorescent probe, in HSA increases by ∼4.5 times when HSA is anchored on the nanoparticle surface, indicating a rise in the loading capacity.

The femtosecond to nanosecond transient absorption results of the SPR peak of the AuNPs reveal the different dynamics involved in the relaxation process after excitation at 460 nm. Three lifetime components were measured and assigned to the electron–electron (∼400 fs), electron–phonon (∼2.0 ps) and phonon–phonon (200–250 ps) interactions. Albumin-coated AuNPs produces similar dynamics which indicates the chemical and physical stability of the AuNPs upon bioconjugation. The presence of FL did not change the surface dynamics of the AuNPs. The presence of FL suppresses the transient absorption of the AuNPs in the blue spectral region due to the large extinction coefficient of FL in the region 470–490 nm. Chemical denaturation of HSA was performed by measuring the spectral shift in the Trp214 fluorescence peak and the rise of the Tyr fluorescence at 308 nm. Unfolding started at [GdnHCl] ≥ 2.0 M and is complete at [GdnHCl] = 6.0 M. HSA anchored on the nanoparticle surface shows more resistance to the unfolding effect, indicating the stability of the native form of HSA on the AuNP surface. Upon a complete unfolding of domain IIA, a larger red shift in the Trp214 fluorescence was observed for the HSA–AuNP complex. This observation indicates that HSA is still attached to the AuNP surface after unfolding, whereas Trp214 is facing the bulk water and the hydration shell rather than the core of the nanoparticle.

The current study is anticipated to contribute towards a better understanding of the physical and dynamical properties of protein-coated noble metal nanoparticles which will help in optimizing their properties for critical applications. Bioimaging, therapy, and drug delivery are among the most important applications of these systems in recent years.

## Conflicts of interest

There are no conflicts to declare.

## Supplementary Material

RA-008-C8RA00006A-s001
