# Supplementary material for: Spectroscopic characterization of the warfarin drug-binding site of folded and unfolded human serum albumin anchored on gold nanoparticles: effect of bioconjugation on the loading capacity
Source: RSC Adv. 2018 Feb 16;8(14):7523–32. doi: 10.1039/c8ra00006a (PMC9078392; doi:10.1039/c8ra00006a)
Supplement: RA-008-C8RA00006A-s001 [file RA-008-C8RA00006A-s001.pdf]

Electronic Supplementary Material (ESI) for RSC Advances.
This journal is © The Royal Society of Chemistry 2018

SUPPLEMENTARY INFORMATION

Spectroscopic characterization of the warfarin drug-binding site of folded and unfolded human serum albumin anchored on gold nanoparticles: effect of bioconjugation on the loading capacity

Saba A.J. Sulaiman,[a] *Tanjjal Bora*[b] *and Osama K. Abou-Zied**[a]

[a] Department of Chemistry, Faculty of Science, Sultan Qaboos University, P.O. Box 36, Postal Code 123, Muscat, Sultanate of Oman. abouzied@squ.edu.om
[b] Nanotechnology Research Center, Sultan Qaboos University, P.O. Box 17, Postal Code 123, Muscat, Sultanate of Oman

Crystal Structure of HSA:

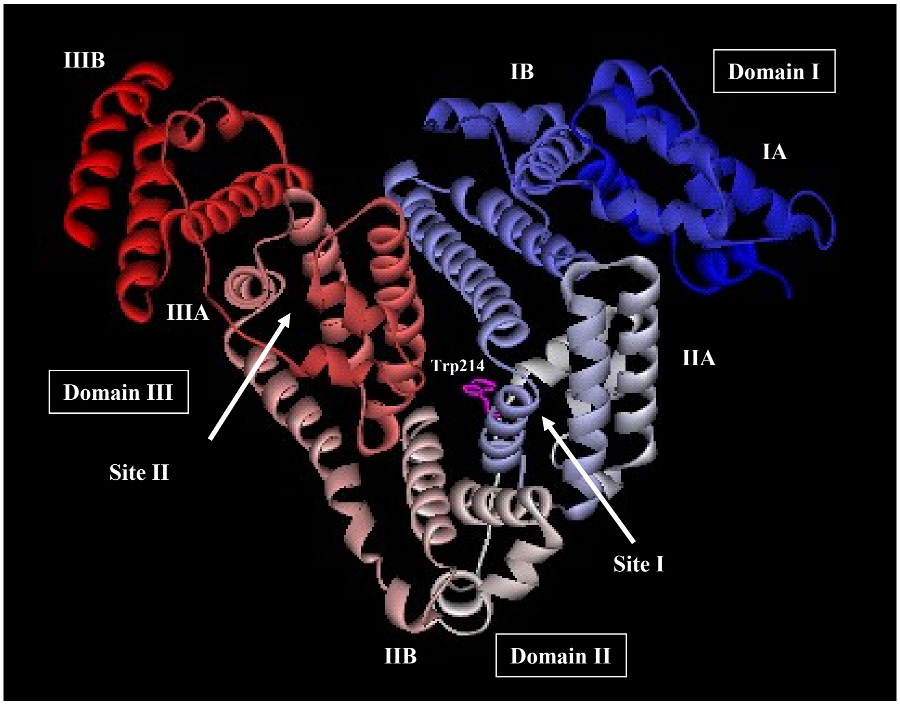

Fig S1 The crystal structure of HSA and the locations of domain-binding sites. The locations of hydrophobic binding sites (Site I and Site II) are shown. The position of tryptophan residue (Trp214) in subdomain IIA is shown. The structure was obtained from the Protein Data Bank (ID code 1ha2).[1]

TEM image of AuNPs:

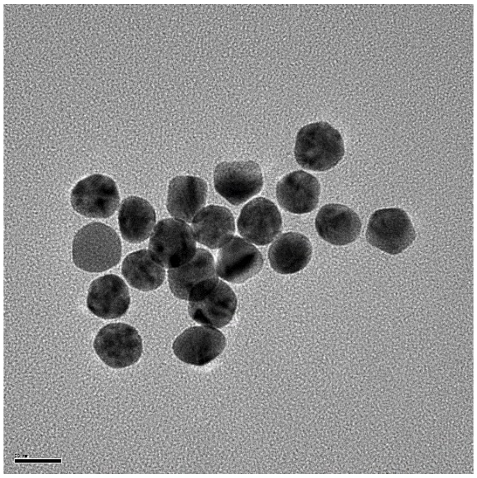

Fig. S2 TEM image of the AuNPs on a larger scale.

Energy dispersive spectra of the gold nanoparticles:

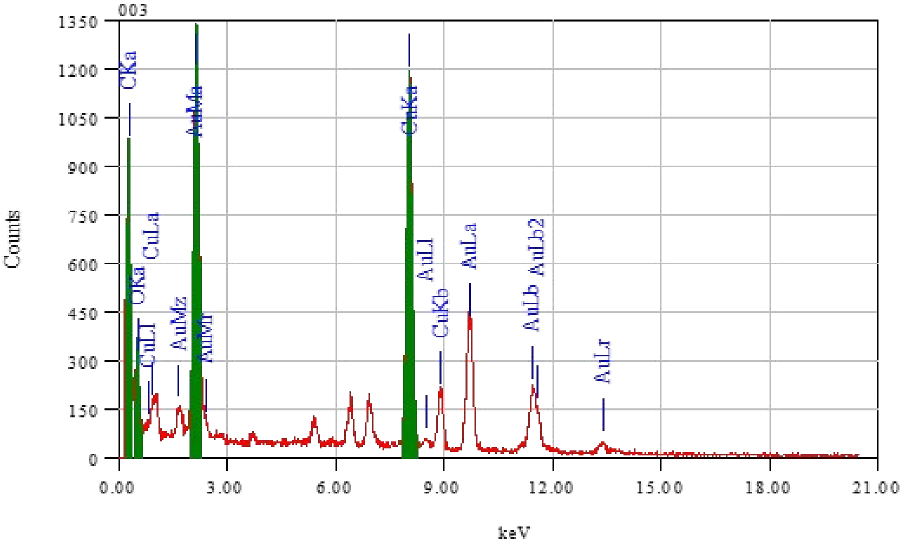

Fig. S3 Energy dispersive spectra of the AuNPs.

Förster's resonance energy transfer:

The distance between the donor (Trp214) and acceptor (AuNP) can be calculated according to Förster's theory for resonance energy transfer (FRET).[2-4] The efficiency of energy transfer, E, is related to the distance (R_{DA}) between the donor and acceptor by

$$E = \frac{R_0^6}{R_0^6 + R_{DA}^6} = 1 - \left(\frac{F}{F_0}\right) \quad (1)$$

where R_0 is the Förster distance (critical distance) when the efficiency of energy transfer is 50%. F and F_0 are the fluorescence intensities of HSA in the presence and absence of the ligand, respectively. The value of R_0 can be calculated from

$$R_0 = 0.211(\kappa^2 d^{-4} \phi_D J)^{1/6} \quad (2)$$

where κ^2 is the spatial orientation factor between the emission dipole of the donor and the absorption dipole of the acceptor, d is the refractive index of the medium, ϕ_D is the fluorescence quantum yield of the donor, and J is the overlap integral of the fluorescence emission spectrum of the donor and the absorption spectrum of the acceptor and is given by

$$J = \sum F(\lambda)\varepsilon(\lambda)\lambda^4 \frac{\Delta\lambda}{\sum F(\lambda)\Delta\lambda} \quad (3)$$

where $F(\lambda)$ is the fluorescence intensity of the donor at wavelength λ, and $\varepsilon(\lambda)$ is the molar absorption coefficient of the acceptor at wavelength λ.

J can be evaluated by integrating the overlapped portion of the spectra in Figure S4. If both the donor and acceptor are tumbling rapidly and are free to assume any orientation, then the dipole orientation factor, κ^2, can adapt the value 2/3, which is the value of the donors and acceptors that randomize by rotational diffusion prior to energy transfer.[3] The value of κ^2 represents a major factor in the analysis of the energy transfer efficiencies. This factor reflects the angle between the emission transition dipole of the donor and the transition absorption dipole of the acceptor. Depending upon the relative orientation of the transition moments of the donor and acceptor. This factor can range from 0 to 4. It is assumed that if both donor and acceptor rotate rapidly compared to the donor excited state lifetime, κ^2 is expected to approach its average value of 2/3. For head-to-tail parallel transition dipoles or antiparallel dipoles (nonrestricted energy transfer) κ^2 = 4, and for parallel dipoles κ^2 = 1.[5-8] Since the sixth root is taken to calculate the distance, variation of κ^2 from 1 to 4 results in a 26% change in R_{DA}. Compared to κ^2 = 2/3, the calculated distance can be in error by no more than 35%. However, if the dipoles are oriented perpendicular to one another (i.e no energy transfer), κ^2 = 0, which would result in serious errors in the calculated distance. Herein, we considered the value of κ^2 to be 0.476 where it is assumed that a range of static donor-acceptor orientations are present and do not change during the lifetime of the excited state.[5]

In the present case, d = 1.33 and ϕ_D = 0.118. Using the aforementioned parameters, we calculated the values summarized in Table S2. For the current system, the concentration of the AuNPs was 0.78 nM based on the particle diameter dimensions[9] and that of HSA was 4.0 µM (mono-dispersed layer coverage).[10]

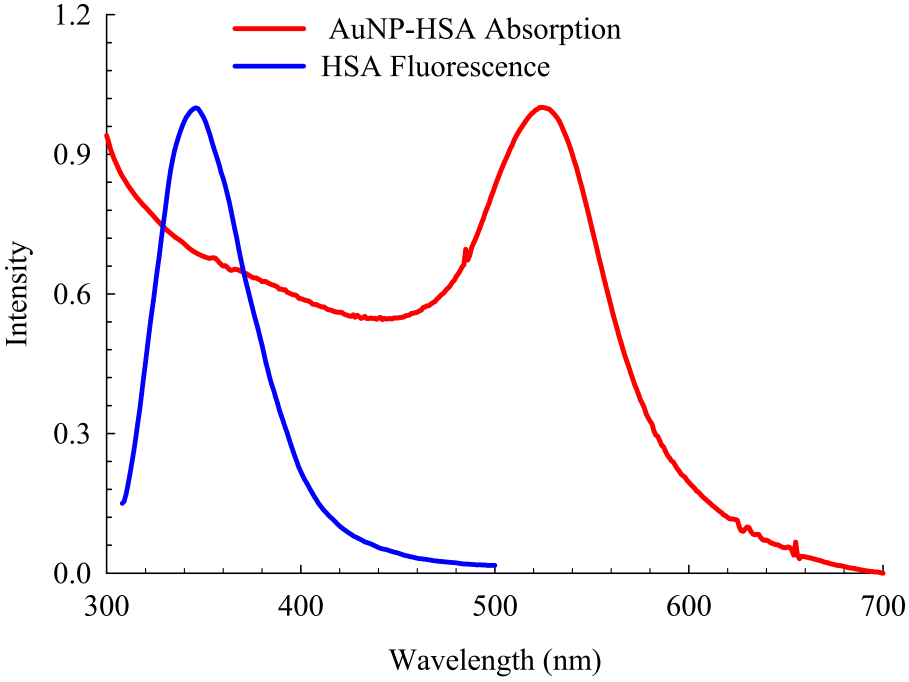

Fig. S4 Overlap of the fluorescence spectrum of HSA (λ_{ex} = 295 nm) with the absorption spectrum of AuNPs as indicated in the graph. For the AuNPs, ε = (8.78 ± 0.06) x 10^8 $M^{-1}cm^{-1}$ for ~ 25 nm in diameter.[9]

Table S1. Calculated parameters for the AuNP-HSA complex using FRET theory

Efficiency of Energy Transfer (%*E*)	67
Donor-Acceptor Critical Distance (R_0 (nm))	20.4
Donor-Acceptor Apparent Distance (R_{DA} (nm))	18.1
Energy Transfer Rate Constant (k x 10^{-8} (s^{-1}))	4.1

Fluorescence lifetimes of HSA:

Decay transients of HSA are shown in Figure S5. The Trp214 residue in HSA is the main source of the fluorescence decay. Two lifetime components were extracted from each decay transient that reflect the dynamics of Trp214. Previous investigations have found that the lifetime of Trp214 in normal HSA is heterogeneous. In most cases this heterogeneity was analyzed in terms of discrete exponential decays. Previously reported lifetimes of native HSA show two main components with a long decay in the range 5.5-8.0 ns and a short component of 1.0-3.5 ns.[5,11-21] The fluorescence decay transients of HSA in the absence and presence of AuNPs are shown in Figure S5. Our results for the decay of HSA are summarized in Table S2. The best fits for HSA were obtained using a biexponential function. The two time constants of HSA are 5.32 ns and 1.31 ns. Our results agree well with the abovementioned values. Upon coating the AuNPs with HSA, we observed no change in the lifetimes of HSA as shown in Figure S5and Table S2.

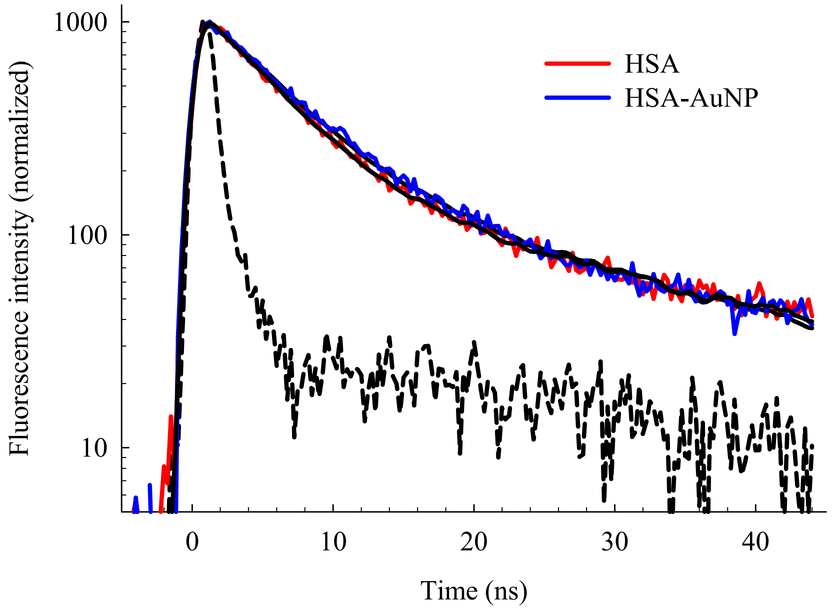

Fig. S5 Fluorescence decay transients of 4 μM HSA in the absence and presence of AuNPs after excitation at 295 nm and observation through a long-path filter 320 nm. IRF curve is shown by the dashed line. Black lines represnet the biexponential fits.

Table S2: Fluorescence lifetimes (ns) of HSA in the absence and presence of AuNPs

	τ_1	τ_2	τ_{av}	χ^2
HSA	5.79 ± 0.1 (0.97)	0.94 ± 0.04 (0.03)	5.65	1.2
HSA + AuNPs	5.93 ± 0.05 (0.94)	0.48 ± 0.02 (0.06)	5.60	1.1

[a] Concentration of HSA was 4 μM. Concentration of AuNPs was 0.78 nM. The medium was deionized water of pH 7.2. λ_{ex} = 295 nm. Fluorescence was observed through a long-path filter 320 nm. Relative contributions are listed in parentheses.

Femtosecond transient absorption results:

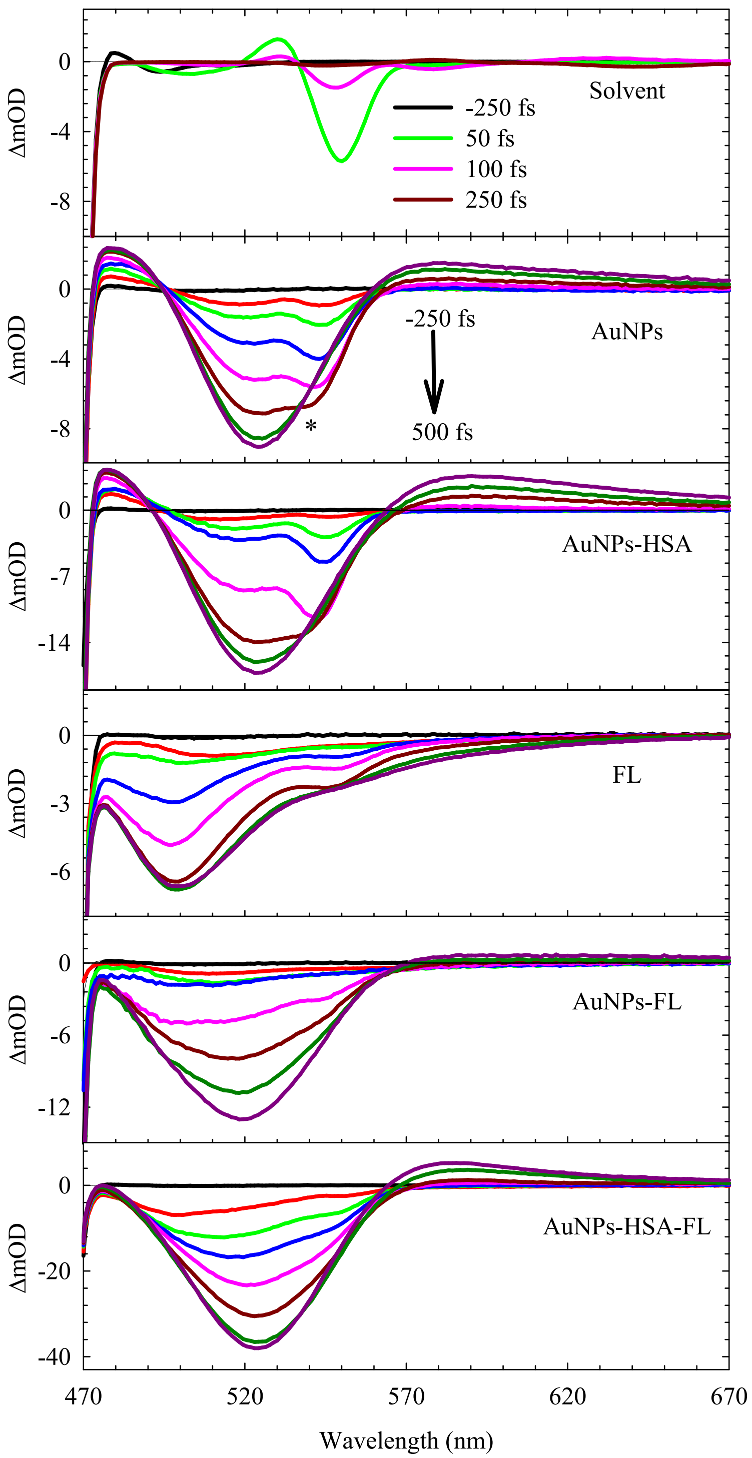

Fig. S6 Femtosecond transient absorption spectra of different systems as indicated in the graph. The dynamics were recorded during the first 500 fs immediately after photoexcitation at 460 nm. * represents Raman scattering due to the solvent. Response due to the solvent is shown at the top.

Unfolding of HSA:

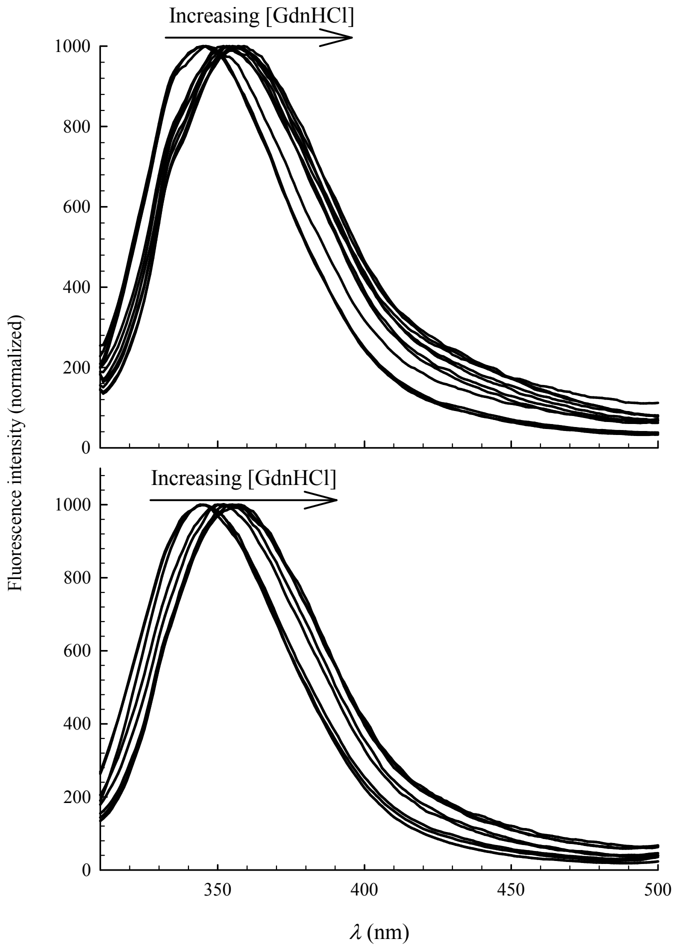

Fig. S7 Selected spectra (normalized) of the fluorescence change of 4 μM HSA as a function of [GdnHCl]. Upper panel: HSA in water, lower panel: HSA−AuNPs complex. λ_{ex} = 295 nm.

References

1 X. M. He and D. C. Carter, Nature, 1992, **358**, 209–215.
2 T. Förster and O. Sinanoglu, *Academic, New York*, 1965, 93–137.
3 T. Förster, Discuss. *Faraday Soc*., 1959, **27**, 7–17.
4 M. Y. Berezin and S. Achilefu, *Chem. Rev.*, 2010, **110**, 2641–2684.
5 J Lakowicz, *Principles of Fluorescence Spectroscopy,* Springer, 2006**.**
6 R. E. Dale, J. Eisinger and W. E. Blumberg, *Biophys J.* 1979, **26**,161–94.
7 R. E. Dale and J. Eisinger, *Biochemical Fluorescence: Concepts*, 1975, **1**, 115–284.
8 R. E. Dale and J. Eisinger, *Biopolymers*, 1974, **13**, 1573–1605.
9 X. Liu, M. Atwater, J. Wang and Q. Huo, *Colloid. Surf. B*, 2007, **58**, 3–7.
10 S. Goy-López, J. Juárez, M. Alatorre-Meda, E. Casals, V. F. Puntes, P. Taboada and V. Mosquera, *Langmuir*, 2012, **28**, 9113–9126.
11 J. M. Beechem and L. Brand, *Ann. Rev. Biochem.*, 1985, **54**, 43–71.
12 O. K. Abou-Zied and O. I. K. Al-Shihi, *J. Am. Chem. Soc.*, 2008, **130**, 10793–10801.
13 M. K. Helms, C. E. Petersen, N. V. Bhagavan and D. M. Jameson, *FEBS letters*, 1997, **408**, 67–70.
14 D. M. Davis, D. McLoskey, D. J. Birch, P. R. Gellert, R. S. Kittlety and R. M. Swart, *Biophy. Chem.*, 1996, **60**, 63–77.
15 J. R. Lakowicz and I. Gryczynski, *Biophys. Chem.*, 1992, **45**, 1–6.
16 P. Marzola and E. Gratton, *J. Phys. Chem.*, 1991, **95**, 9488–9495.
17 K. VOS, A. HOEK and A. J. VISSER, *Eur. J. Biochem.*, 1987, **165**, 55–63.
18 S. Kasai, T. Horie, T. Mizuma and S. Awazu, *J. Pharm. Sci.*, 1987, **76**, 387–392.
19 G. Hazan, E. Haas and I. Steinberg, *Biochim. Biophys. Acta,* 1976, **434**, 144–153.
20 I. Z. Steinberg, *Annu. Rev. Biochem.*, 1971, **40**, 83–114.
21 W. De Lauder and P. Wahl, *Biochem. Biophys. Res. Commun*, 1971, **42**, 398–404.
